# Tryptophan metabolite 5-methoxytryptophan ameliorates arterial denudation-induced intimal hyperplasia via opposing effects on vascular endothelial and smooth muscle cells

**DOI:** 10.18632/aging.102350

**Published:** 2019-10-09

**Authors:** Chung-Huang Chen, Yen-Chun Ho, Hua-Hui Ho, Li-Yu Liang, Wei-Cheng Jiang, Guan-Lin Lee, Jen-Kuang Lee, Yu-Juei Hsu, Cheng-Chin Kuo, Kenneth K. Wu, Shaw-Fang Yet

**Affiliations:** 1 Institute of Cellular and System Medicine, National Health Research Institutes, Zhunan 35053, Taiwan; 2 Division of Cardiology, Department of Internal Medicine, National Taiwan University Hospital and National Taiwan University College of Medicine, Taipei 10002, Taiwan; 3 Division of Nephrology, Department of Medicine, Tri-Service General Hospital, Taipei 11490, Taiwan; 4 Graduate Institute of Biomedical Sciences, China Medical University, Taichung 40402, Taiwan; 5 Department of Medical Sciences and Institute of Biotechnology, National Tsing Hua University, Hsinchu 30013, Taiwan

**Keywords:** tryptophan metabolite, 5-MTP, restenosis, endothelial cells, vascular smooth muscle cells

## Abstract

Cardiovascular diseases remain the leading cause of morbidity and mortality worldwide, particularly among older adults. Despite the advent of medical technology, restenosis is still an issue after interventional procedures. Tryptophan metabolite 5-methoxytryptophan (5-MTP) has recently been shown to protect against systemic inflammatory responses. This study aimed to investigate the function and mechanisms of 5-MTP in interventional procedure-induced restenosis. We found that after mouse femoral artery denudation with a guide wire, 5-MTP accelerated recovery of endothelium in the denuded area and reduced vascular leakage and intimal thickening. 5-MTP increased endothelial cell proliferation in the denuded arteries and rescued TNF-α-reduced endothelial cell proliferation and migration, likely via maintaining vascular endothelial growth factor receptor 2 activation. In contrast, 5-MTP preserved differentiated phenotype of medial vascular smooth muscle cells (VSMCs) and decreased VSMC proliferation and migration. Furthermore, 5-MTP maintained expression levels of critical transcription factors for VSMC marker gene expressions via attenuated activation of p38 MAPK and NFκB-p65. Our findings uncover a novel protective mechanism of 5-MTP in restenosis. In response to denudation injury, 5-MTP attenuates intimal hyperplasia via concerted but opposing actions on endothelial cells and VSMCs. Taken together, our results suggest that 5-MTP is a valuable therapeutic target for arterial injury-induced restenosis.

## INTRODUCTION

Cardiovascular disease remains the leading cause of death worldwide and the major global burden of disease, particularly among older adults [1, 2]. It is well recognized that aging contributes to the pathogenesis of atherosclerosis [3, 4], therefore population aging further worsens the situation. In the United States, coronary heart disease (CHD) is the number one cause of deaths attributable to cardiovascular disease [5]. The main underlying cause of CHD is atherosclerosis, a vascular disease progressively occludes the lumen of arteries with atherosclerotic lesions [6]. To relieve vessel occlusion, endovascular procedures such as balloon percutaneous transluminal coronary angioplasty (with or without stenting) are essential for the treatment. However, interventional procedures can injure blood vessel wall such as removal of the endothelium and medial layer damage, leading to intimal hyperplasia and restenotic lesion formation [7, 8], which remains a critical issue following interventional procedures. After denudation of endothelium, proliferation and migration of medial vascular smooth muscle cells (VSMCs) contribute to neointima formation [9–11]. Identifying new complementary therapeutic targets for preventing restenosis is still an unmet medical need.

Tryptophan is an essential amino acid and an important source for protein synthesis; its various metabolites participate in diverse physiological functions [12]. Aberrant tryptophan metabolism leads to development of diseases [13]. Patients with cardiovascular disease have increased serum kynurenine/tryptophan ratio. Platelet-derived serotonin (5-hydroxytryptamine) and its receptor signaling links vascular disease and tissue fibrosis [14]. These studies indicate the critical importance of tryptophan metabolism in cardiovascular diseases. Intriguingly, organisms possess defense mechanisms by generating endogenous protective molecules. Indeed, 5-methoxytryptophan (5-MTP), a tryptophan metabolite, protects against tumorigenesis by suppressing inflammatory cyclooxygenase-2 expression [15]. Recent studies show that 5-MTP is a novel class of endothelium-derived protective molecule that defends against excessive systemic inflammatory responses [16] and prevents human umbilical vein endothelial cells (HUVECs) from inflammatory mediator-induced hyperpermeability [17]. A recent study shows that serum 5-MTP levels decrease with progression of chronic kidney disease and treatment with 5-MTP ameliorates renal interstitial fibrosis [18]. We have shown that circulating 5-MTP concentrations are inversely correlated with CHD in humans and 5-MTP attenuates cessation of blood flow-induced vascular remodeling [19], suggesting a protective role of 5-MTP in vascular disease. However, the role of 5-MTP in restenosis after endothelium denudation, which mimics balloon angioplasty, is not known. In this study, we used a neointima formation model by removing endothelium of mouse femoral artery with a guide wire to investigate whether 5-MTP affects intimal hyperplasia following arterial denudation injury and explore the underlying mechanisms.

## RESULTS

### Reduced intimal thickening by 5-MTP 4 weeks following arterial denudation injury

**Figure 1:**
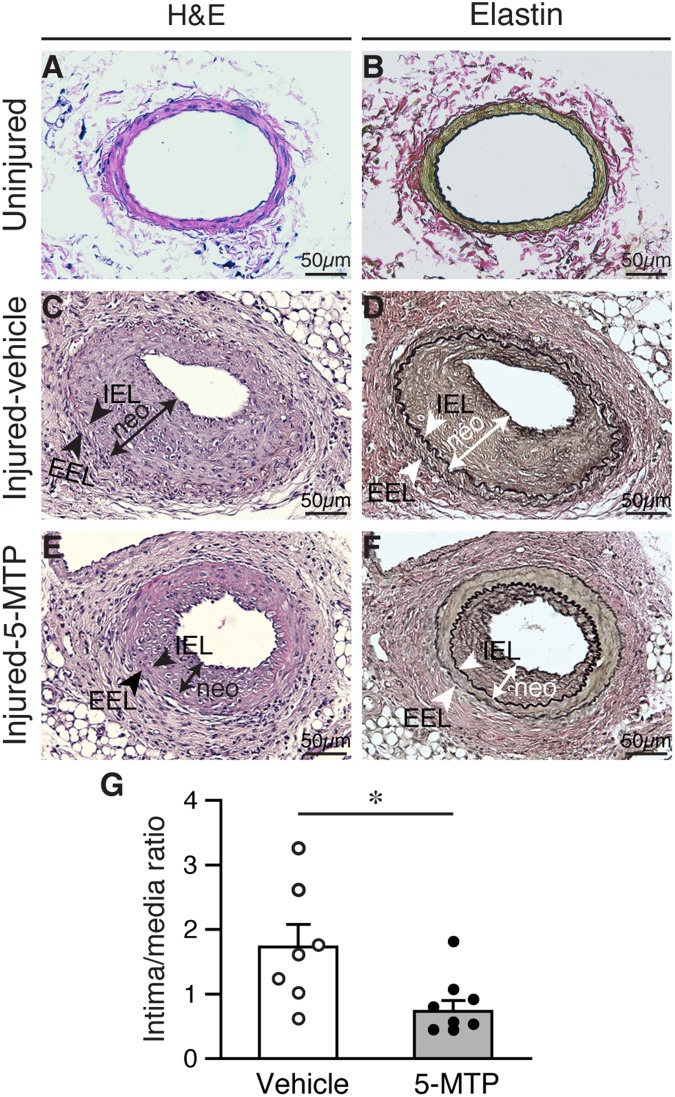
**5-MTP reduces arterial denudation-induced intimal hyperplasia.** Mice were subjected to femoral artery denudation injury and treated with PBS or 5-MTP. Vessels were harvested 4 weeks later. (**A**–**B**) Uninjured vessel cross sections were stained with H&E (**A**) or Verhoeff’s staining for elastin (**B**). (**C**–**F**) Sections from injured arteries from vehicle (**C**–**D**) or 5-MTP-treated (**E**–**F**) mice were stained for H&E (**C** and **E**) or elastin stain (**D** and **F**). Representative sections are shown. Arrowheads indicates internal elastic lamina (IEL) and external elastic lamina (EEL), respectively, while a line with arrowhead at both ends delineates neointima (neo). (**G**) Quantitative morphometric analysis of intimal and medial area and expressed as intima/media ratio in injured vehicle- (1.73±0.35, n=7) and 5-MTP-treated (0.73±0.17, n=9) mice (*p=0.0076 vs. vehicle).

To investigate whether 5-MTP has a role in restenosis, we subjected mice to a restenosis model by denuding femoral arteries and treated with vehicle or 5-MTP. Injured and contralateral uninjured femoral arteries were harvested 4 weeks later for histological analysis. H&E staining of vessel sections showed patent uninjured arteries (Figure 1A). Verhoeff’s staining, to delineate elastin layers, also showed patent uninjured arteries (Figure 1B). Arterial denudation induced robust intimal hyperplasia in femoral arteries from vehicle-treated mice (Figure 1C and 1D). In comparison, 5-MTP-treated mice had smaller neointima (Figure 1E and 1F). Morphometric analysis revealed that 5-MTP significantly decreased intima/media ratio from 1.73±0.35 to 0.73±0.17 (Figure 1G), indicating 5-MTP attenuates intimal hyperplasia after arterial denudation.

### Improved endothelial coverage in injured femoral arteries by 5-MTP treatment

**Figure 2:**
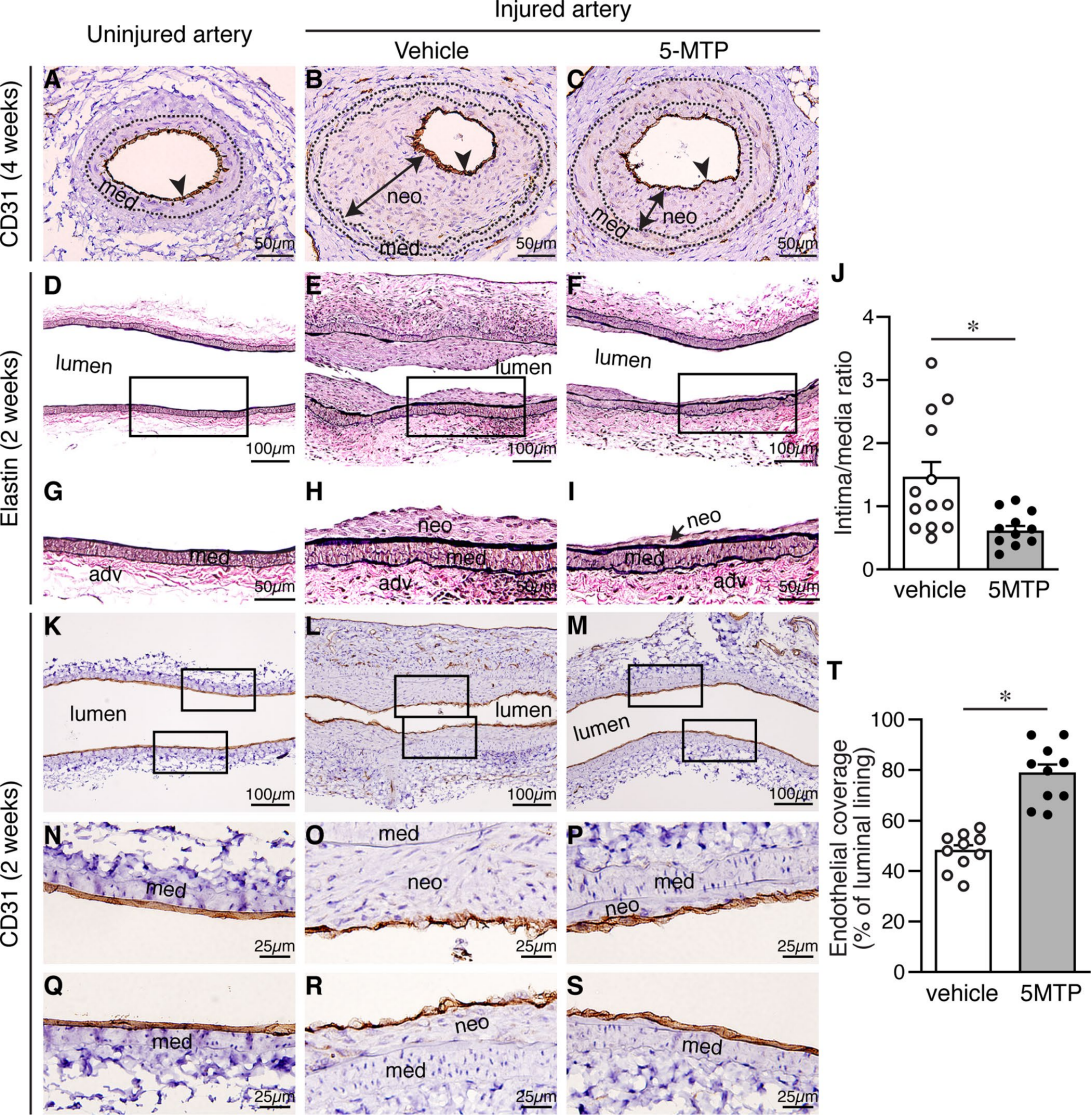
**5-MTP increases endothelial coverage following arterial denudation.** Arteries from uninjured and injured mice were harvested and sectioned for histological analysis. (**A**–**C**) Four weeks after injury, arterial sections from uninjured (**A**) and injured-vehicle (**B**) or injured-5-MTP (**C**) mice were stained with CD31 antibody to delineate endothelium (brown, arrowhead). A line with arrowheads at both ends indicates neointima (neo). med, media. (**D**–**F**) Two weeks after injury, longitudinal arterial sections from uninjured (**D**), injured vehicle-treated (**E**), and injured 5-MTP-treated (**F**) mice were stained with Verhoeff’s elastin stain. (**G**–**I**) Magnified areas from boxed area in (**D**–**F**), respectively. (**J**) Quantitative morphometric analysis of intima/media ratio in vehicle (1.45±0.24, n=13) and 5-MTP-treated (0.60±0.09, n=12) mice (*p=0.0056 vs. vehicle). (**K**–**M**) CD31 immunohistochemistry to identify endothelial cells (brown). Artery from uninjured (**K**), Injured vehicle-treated (**L**), and injured 5-MTP-treated (**M**) mice. (**N**–**P**) Magnified areas from upper box of (**K**–**M**), respectively. (**Q**–**S**) Magnified areas from lower box of (**K**–**M**), respectively. (**T**) Quantitation of endothelial coverage in vehicle (47.9±2.3%, n=10) and 5-MTP-treated mice (78.6±3.7%, n=10; *p=0.000001).

Recovery of endothelium is critical in vessel repair and protecting arteries against further injury. To determine whether the differences in neointimal size were due to differences in reendothelialization, we examined the extent of endothelial coverage in injured vessels. In normal uninjured vessels, CD31 staining delineates a tight layer of endothelium (Figure 2A). Four weeks after injury, we detected near complete endothelial coverage in both vehicle and 5-MTP group (Figure 2B–2C). It has been reported that reendothelialization is near complete 3 weeks following endoluminal injury [20]. We thus examined neointima formation and endothelial coverage at an earlier 2-week time point. To have a better assessment of the whole artery, we sectioned vessels longitudinally. Elastin stain revealed that compared with uninjured arteries (Figure 2D and 2G), substantial neointima was present in vehicle-treated mice 2 weeks after denudation (Figure 2E and 2H). In comparison, neointima size was much smaller in 5-MTP-treated arteries (Figure 2F and 2I). 5-MTP significantly decreased intima/media ratio from 1.45±0.24 to 0.60±0.09 (Figure 2J). We next examined endothelial coverage by CD31 staining. In contrast to a tight endothelial layer of uninjured artery (Figure 2K, 2N, and 2Q), 2 weeks following denudation CD31 staining appeared discontinuous with intermittent negative areas (Figure 2L, 2O, and 2R). In comparison, 5-MTP-treated group had improved endothelial coverage (Figure 2M, 2P, and 2S). Quantitative analysis showed that 5-MTP significantly increased endothelial coverage on the luminal surface (Figure 2T). These results indicate that 5-MTP increases endothelial coverage on the luminal surface after arterial injury.

### Attenuation of vascular leakage following arterial injury by 5-MTP

**Figure 3:**
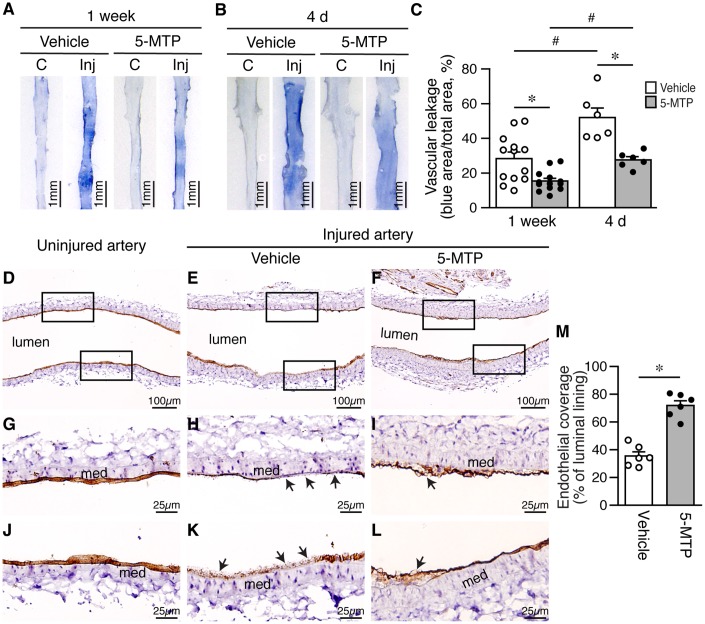
**5-MTP reduces vascular leakage and increases endothelial coverage after arterial denudation.** (**A**–**B**) Vascular leakage was assessed by Evans blue dye perfusion after injury. (**C**) contralateral uninjured right femoral artery; Inj, injured left femoral artery. (**A**) One week after injury (vehicle, n=13; 5-MTP, n=13). (**B**) Four days after injury (vehicle, n=10; 5-MTP, n=8). (**C**) Quantitation of vascular leakage. *p<0.004 vs. vehicle; ^#^*p*<0.003 vs. 1 week. (**D**–**F**) CD31 immunohistochemistry for ECs (brown) of arteries from uninjured (**D**), injured-vehicle (**E**), and injured-5-MTP mice (**F**) 1 week after injury. (**G**–**L**) Magnified areas from upper and lower box of (**D**–**F**), respectively. Arrows, CD31-negative areas; med, media. (**M**) Quantitation of endothelial coverage 1 week after injury (*p<0.00002 vs. vehicle).

Disruption of endothelial integrity compromises barrier function and leads to vascular leakage. Thus, we next examined barrier function of femoral arteries after injury with Evan’s blue dye. One week after denudation, strong blue staining, indicative of vascular leakage, was observed in vehicle-treated injured arteries, compared with control contralateral artery without injury (Figure 3A). In comparison, 5-MTP reduced blue staining intensity in injured artery (Figure 3A). Quantitative analysis showed that 5-MTP reduced vascular leakage (Figure 3C). At an early time point 4 d after injury, barrier function assays revealed that more intense blue staining was observed in vehicle-treated injured arteries (Figure 3B), indicative of worse vascular leakage than 1 week (Figure 3C, vehicle, white bars). Compared with vehicle, 5-MTP significantly decreased vascular leakage at 4 d (Figure 3B and 3C). These results indicate that 5-MTP attenuates vascular leakage starting at early time points following arterial injury.

### 5-MTP enhances reendothelialization and endothelial proliferation after denudation injury

Because 5-MTP attenuated vascular leakage after denudation, we next investigated whether 5-MTP enhances repair of endothelium by examining endothelial coverage after injury. At 1 week, compared with uninjured arteries (Figure 3D, 3G, and 3J), vehicle-treated injured arteries had large CD31-negative area (Figure 3E, 3H, and 3K; arrows) whereas 5-MTP group showed smaller CD31-negative area on the luminal surface (Figure 3F, 3I, and 3L). Quantitation revealed that 5-MTP significantly increased endothelial coverage 1 week after injury from 35.5±3.1% of vehicle to 71.9±3.5% (Figure 3M), indicating increased endothelial repair by 5-MTP.

**Figure 4:**
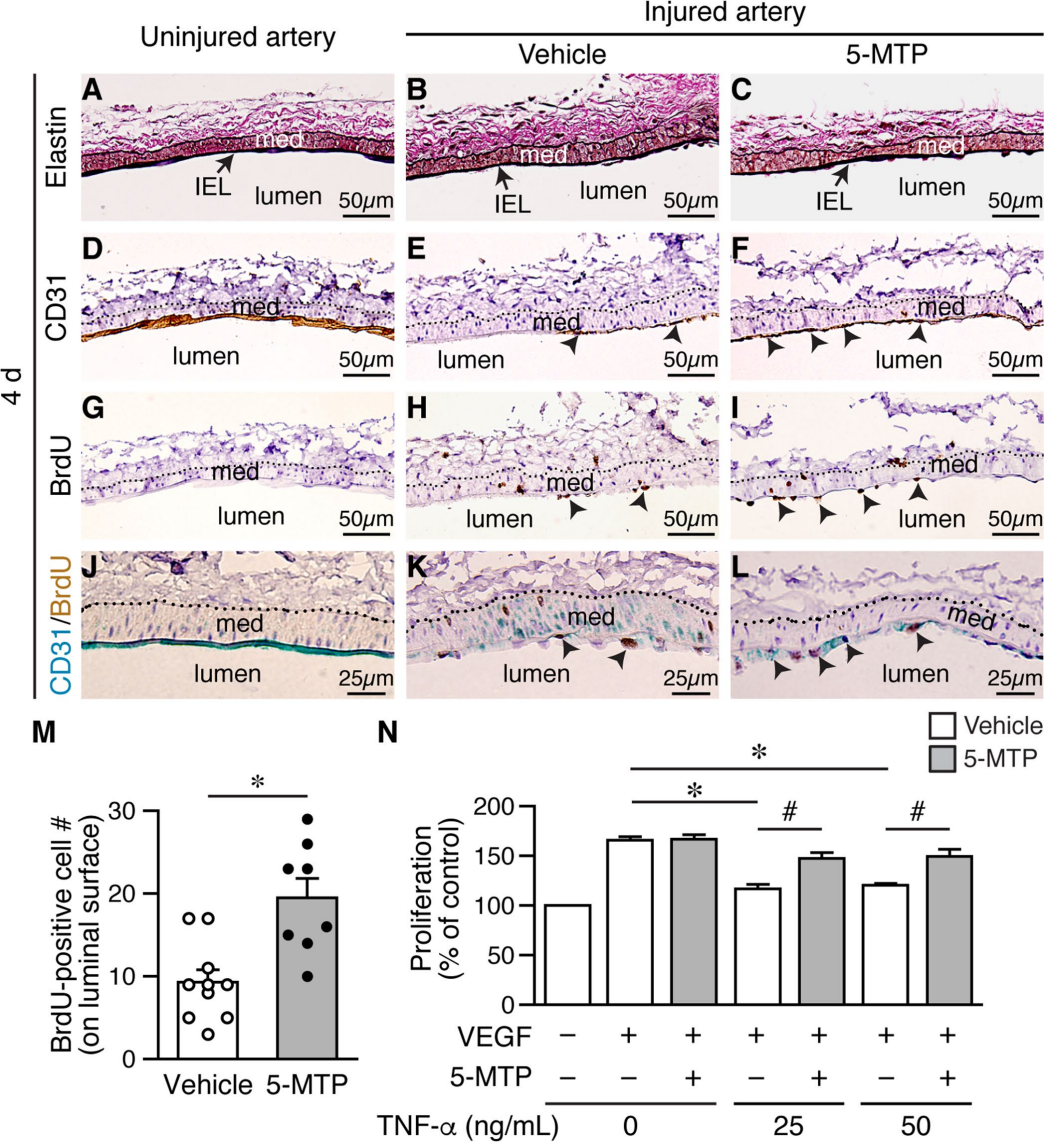
**5-MTP increases endothelial proliferation following arterial denudation. Femoral arteries were harvested 4 d after injury.** (**A**–**C**) Elastin stain of longitudinal vessel sections. Arrows, internal elastic lamina (IEL); med, media. (**D**–**F**) CD31 staining for ECs. Arrowheads, CD31-positive brown cells. (**G**–**I**) BrdU immunostaining for proliferating cells. Arrowheads, BrdU-positive brown nuclei. Dashed line, external elastic lamina. (**J**–**L**) Double immunostaining of CD31 (blue) and BrdU (brown). Arrowheads, double-positive cells. (**M**) Quantitation of BrdU-positive cells on the luminal surface of arteries from vehicle (n=10) and 5-MTP-treated mice (n=8; *p<0.002). (**N**) TNF-α decreased HUVEC proliferation induced by VEGF (n=4 each, *p<0.001). 5-MTP rescued TNF-α-decreased endothelial proliferation (n=4 each, ^#^*p*<0.008).

To confirm the effect of 5-MTP on reendothelialization following denudation, we harvested vessels 4 d after injury. Elastin staining revealed intact internal elastic lamina (IEL) in uninjured and injured arteries (Figure 4A-4C), indicating denudation injury per se did not damage IEL. Compared with a complete layer of CD31-positive staining in uninjured artery (Figure 4D), there was only limited CD31-positive area on the luminal surface of injured vehicle-treated artery (Figure 4E, arrowheads). Interestingly, a larger area of CD31-positive staining was observed in 5-MTP-treated injured artery (Figure 4F, arrowheads), suggesting more endothelial cells. We then performed BrdU incorporation assays on adjacent sections to assess whether 5-MTP promoted endothelial cell proliferation to repair denuded area. No BrdU-positive cells were detected in uninjured artery (Figure 4G) while few positive cells were found on the luminal surface of vehicle-treated injured artery (Figure 4H, arrowheads). Importantly, significantly more BrdU-positive cells were detected on the luminal surface of 5-MTP-treated injured artery (Figure 4I, arrowheads). Quantitative analysis showed that 5-MTP treatment increased 2.1-fold of BrdU-positive cell number (Figure 4M). To demonstrate unequivocally that proliferating cells are endothelial cells, we performed double immunostaining with BrdU and CD31 antibodies. Uninjured arteries stained positive for CD31 (blue) on luminal surface while no BrdU-positive (brown) staining was observed (Figure 4J). Very few double-positive cells were detectable (2.0±1.0/section, n=4) in vehicle-treated injured arteries (Figure 4K). In contrast, many double-positive cells with blue staining surrounding brown nucleus were detected (12.3±6.1/section, n=4; *p*=0.02 vs. vehicle) (Figure 4L, arrowheads). These data support the notion that 5-MTP enhances endothelial proliferation following injury.

Signaling network initiated by vascular endothelial growth factor (VEGF) and VEGF receptor 2 (VEGFR2) leads to endothelial cell proliferation [21]. However, proinflammatory cytokines such as TNF-α and IL-1β are released in the vessel wall after injury and TNF-α is known to decrease VEGF-VEGFR2-induced endothelial proliferation. We therefore examined whether 5-MTP has an effect on TNF-α-decreased endothelial proliferation. It has been shown that TNF-α treatment of up to 100 ng/mL does not affect HUVEC viability [22]. We thus treated serum-starved HUVECs with VEGF in the presence of different concentrations of TNF-α and measured proliferation. VEGF increased HUVEC proliferation while TNF-α at 25 and 50 ng/mL significantly decreased proliferation induced by VEGF (Figure 4N, white bars). In contrast, although 5-MTP did not affect VEGF-induced proliferation in the absence of TNF-α it rescued TNF-α-decreased endothelial proliferation at both 25 and 50 ng/mL (Figure 4N, gray bars). These results support the concept that 5-MTP promotes barrier function and reendothelialization in part through increased endothelial proliferation.

### 5-MTP rescues endothelial cell migration and VEGFR2 activation after inflammatory cytokine stimulation

**Figure 5:**
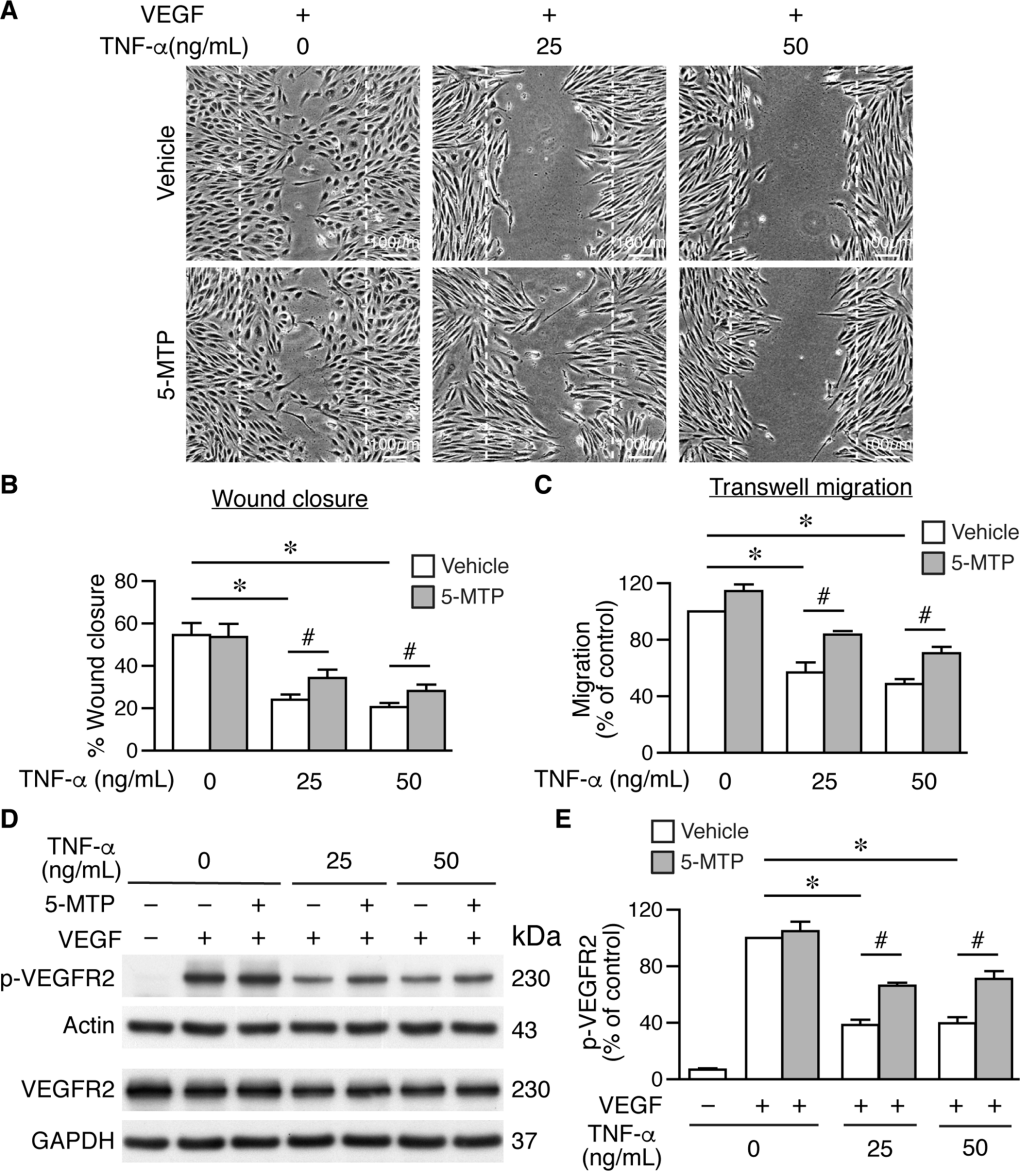
**5-MTP rescues TNF-α-decreased endothelial cell migration and VEGFR2 activation.** Wound healing assays were performed using HUVECs and wound closure evaluated at 0 and 18 h after wounding. (**A**–**B**) Wound closure and quantitation at 18 h (n=4 each group). Dashed lines, wound margins. VEGF promoted cellular migration into wound area. TNF-α decreased VEGF-induced wound closure in the absence of 5-MTP (*p<0.002 vs. control). 5-MTP rescued TNF-α-decreased wound closure (^#^*p*<0.04). (**C**) Transwell migration assays were performed using growth-arrested HUVECs (n=4 each group). TNF-α reduced migration (*p<0.0001) while 5-MTP rescued TNF-α-decreased migration (^#^*p*<0.01). (**D**) HUVECs were stimulated with VEGF for 15 min for Western blotting to detect phosphorylated and total VEGFR2. (**E**) Quantitation of p-VEGFR2 (n=4 per group, *p=0.0001 and ^#^*p*<0.005).

In addition to proliferation, cellular migration also contributes to regeneration of endothelium. To further investigate the mechanisms underlying enhanced reendothelialization by 5-MTP, we examined the effect of 5-MTP on endothelial cell migration. Serum-starved HUVECs were treated with or without 5-MTP, followed by different concentrations of TNF-α, and then stimulated with VEGF for scratch wound assays. In the absence of VEGF, only a few cells migrated into the wound area with or without 5-MTP after 18 h (Supplementary Figure 1A). VEGF promoted substantial cellular migration into wound area regardless of 5-MTP (Supplementary Figure 1A and Figure 5A) whereas TNF-α at 25 and 50 ng/mL decreased VEGF-induced wound closure (Supplementary Figure 1B and Figure 5A-5B). Although 5-MTP treatment in the absence of TNF-α did not affect wound closure, 5-MTP rescued TNF-α-reduced wound closure at both 25 and 50 ng/mL (Supplementary Figure 1B and Figure 5A–5B). To exclude contribution of proliferation in scratch wound repair, we treated HUVECs with mitomycin C to arrest growth and performed transwell migration assays. Consistent with scratch wound results, 5-MTP significantly rescued TNF-α-decreased cellular migration (Figure 5C). These data confirm that 5-MTP rescues endothelial cell migration under pathological conditions.

VEGFR2 phosphorylation in the VEGF-VEGFR2 signaling pathway plays a critical role in endothelial cell proliferation and migration [23–25]. We first evaluated whether 5-MTP has any effect on VEGFR2 phosphorylation. Vehicle or 5-MTP alone did not induce VEGFR2 phosphorylation; in contrast, VEGF induced a robust VEGFR2 phosphorylation (Supplementary Figure 2). We then examined whether 5-MTP affected VEGF-mediated VEGFR2 activation after TNF-α stimulation. VEGF induced VEGFR2 phosphorylation while 5-MTP did not affect this phosphorylation in the absence of TNF-α (Figure 5D–5E). Interestingly, TNF-α decreased VEGF-induced VEGFR2 phosphorylation while 5-MTP significantly rescued the reduction of phosphorylation by TNF-α (Figure 5D–5E). Together, these results indicate that 5-MTP might exert its endothelial protection by sustaining VEGFR2 activation in the setting of inflammatory conditions. NFκB signaling is a well-known pathway downstream of TNF-α, we next examined whether 5-MTP has a suppressive effect on TNF-α-induced NFκB activation in endothelial cells. Interestingly, 5-MTP suppressed TNFα-induced p38 activation but did not inhibit NFκB activation in HUVECs (Supplementary Figure 3). This result is consistent with our previous finding that 5-MTP suppresses inflammatory mediator IL-1β-induced activation of p38 but not NFκB [19], suggesting the protective effect of 5-MTP in endothelial cells is not mediated through NFκB signaling.

### Treatment of 5-MTP decreases VSMC proliferation in response to arterial denudation injury

**Figure 6:**
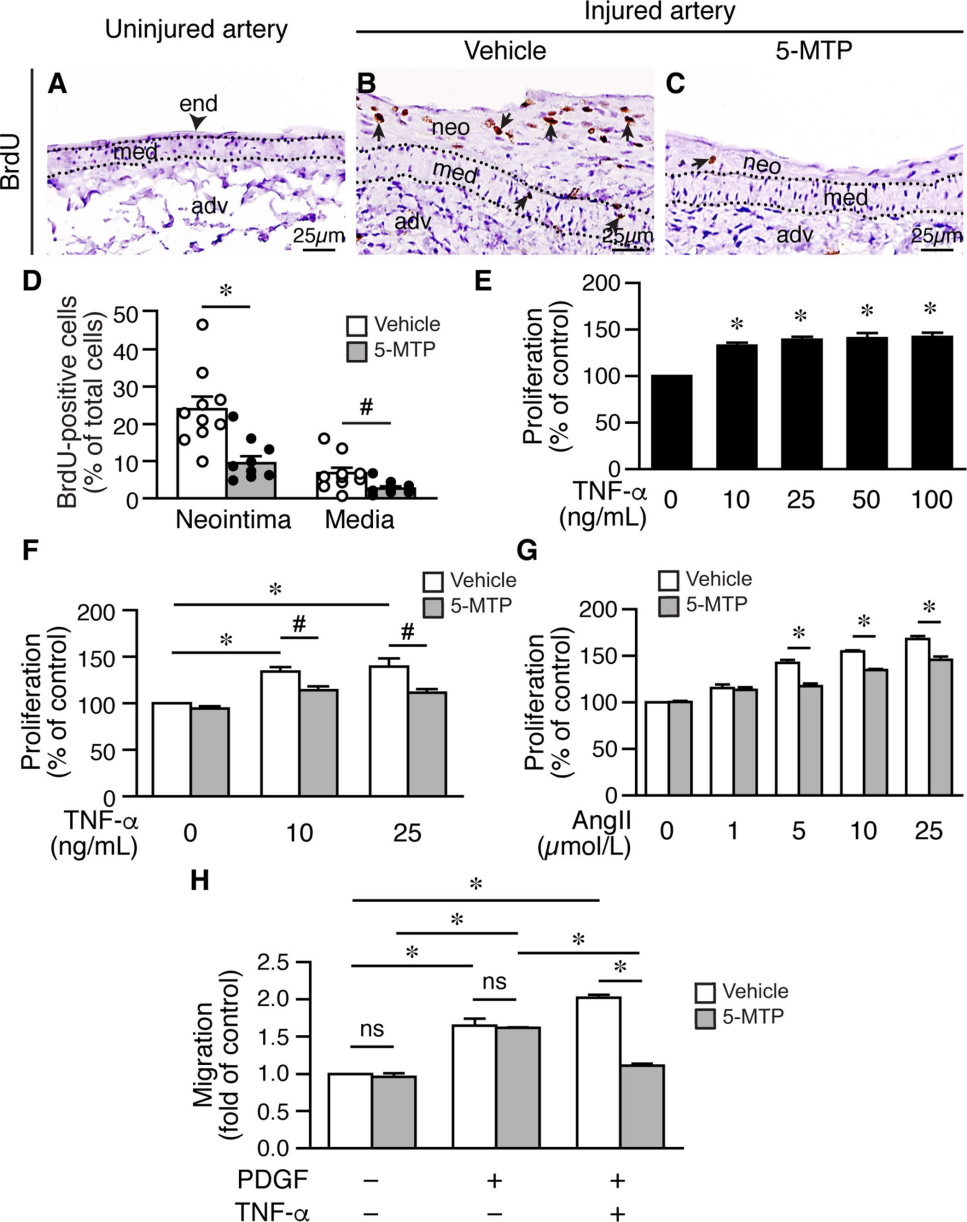
**5-MTP decreases VSMC proliferation following arterial injury.** (**A**–**C**) Femoral arteries were harvested 2 weeks after injury. Arterial sections were immunostained with BrdU antibody (brown) to detect proliferating cells. Arrowhead indicates endothelium (end) and arrows indicate BrdU-positive brown nuclei. adv, adventitia; med, media; neo, neointima. (**D**) BrdU-positive cells were quantified and expressed as % of total cells in the neointima or media. 5-MTP decreased BrdU incorporation in the neointima (n=10 each; *p<0.002) and in the media (n=10 each; #*p*<0.02). (**E**) Serum-starved VSMCs were treated with TNF-α for 24 h, proliferation was then measured and normalized to control without TNF-α (n=3, *p<0.01 vs. control). (**F**) Serum-starved VSMCs in the presence of vehicle or 5-MTP were treated with TNF-α for 24 h, proliferation was then measured and normalized. TNF-α increased VSMC proliferation (n=3 each, *p<0.02). 5-MTP mitigated TNF-α-induced proliferation (n=3 each, *p=0.05). (**G**) VSMCs were treated as in (**F**), stimulated without or with different concentrations of angiotensin II (AngII) for 24 h. Cell proliferation was then measured as in (**F**). n=3 each, *p<0.005. (**H**) VSMCs were treated as in (**F**), stimulated with or without TNF-α for 24 h, and then transwell migration assays performed. Cells migrated through membranes were quantified after 4 h. The migrated cell number of vehicle control without TNF-α treatment and without PDGF-BB as a chemoattractant was set as 1. 5-MTP had no effect on migration under this condition. TNF-α increased VSMC migration (n=3, *p<0.002) while 5-MTP decreased TNF-α-induced migration (n=3, #*p*=0.001). ns, no significance.

Both endothelium and the underlying medial layer play critical roles in repairing injured blood vessels. Following vessel injury, VSMCs in the media become proliferative for the purpose of repairing vessel wall—the response-to-injury hypothesis [26, 27]. However, exaggerated proliferation and migration of VSMCs from media into intimal space contribute to neointima formation and vessel occlusion. Because 5-MTP decreased intimal hyperplasia, we wanted to determine whether 5-MTP decreases VSMC proliferation after denudation. We harvested vessels 2 weeks after denudation and assessed proliferation by BrdU incorporation in the arteries. Compared with uninjured arteries without BrdU incorporation (Figure 6A), many BrdU-positive cells were present in the neointima and media of injured vehicle-treated arteries (Figure 6B). In contrast, only a few BrdU-positive cells were detected in 5-MTP-treated arteries (Figure 6C). Quantitation results showed that 5-MTP significantly decreased BrdU incorporation in the neointima and in the media (Figure 6D). In primary VSMCs, TNF-α induced VSMC proliferation (Figure 6E). In the absence of TNF-α, 5-MTP alone did not affect proliferation (Figure 6F). On the other hand, 5-MTP significantly mitigated TNF-α-induced VSMC proliferation (Figure 6F). We have previously shown that 5-MTP reduces IL-1β- but not PDGF-BB-induced VSMC proliferation [19]. We thus next examined the effect of 5-MTP on angiotensin II, which is important in neointimal formation. In the absence of angiotensin II, 5-MTP did not affect proliferation (Figure 6G). Intriguingly, 5-MTP significantly reduced angiotensin II-increased VSMC proliferation at 5, 10, and 25 μmol/L (Figure 6G). Taken together, it is likely that 5-MTP may preferentially suppress inflammatory mediator-induced VSMC proliferation.

Migration contributes significantly to intimal hyperplasia, we thus assessed whether 5-MTP affects TNF-α-induced VSMC migration. In the absence of PDGF as a chemoattractant, 5-MTP had no effect on cellular migration of VSMCs without TNF-α treatment (Figure 6H). In the presence of chemoattractant PDGF, both vehicle and 5-MTP significantly increased cellular migration and to a similar degree (Figure 6H). TNF-α treatment further increased cellular migration; importantly, 5-MTP reduced approximately 50% of TNF-α-increased VSMC migration (Figure 6H). To exclude the possibility that this was due to a toxic effect of 5-MTP and TNF-α, we performed viability assays. The results revealed that 5-MTP did not affect VSMC viability in the absence or presence of TNF-α (Supplementary Figure 4).

### 5-MTP attenuates downregulation of smooth muscle markers following arterial denudation

**Figure 7:**
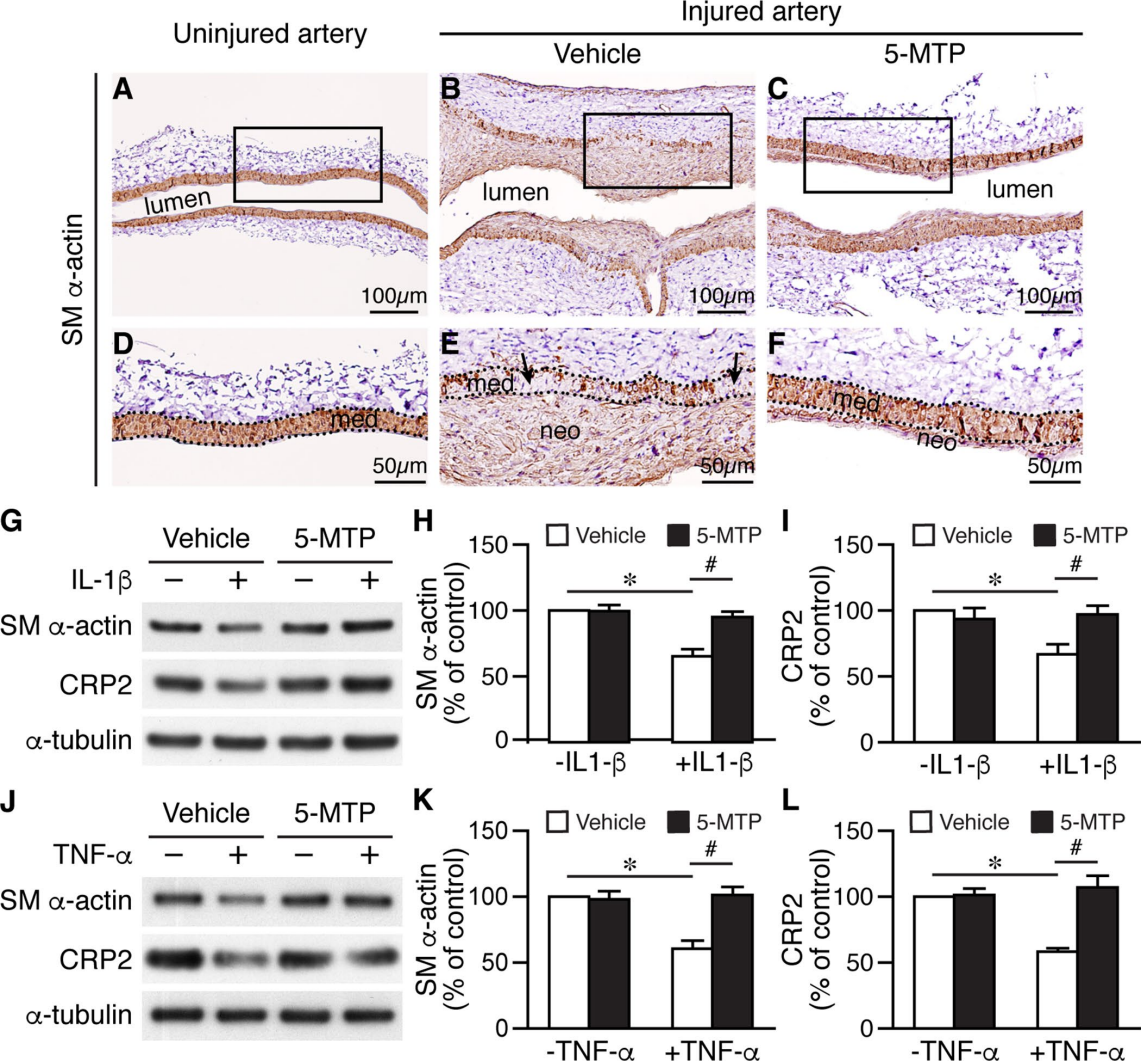
**5-MTP prevents smooth muscle marker downregualtion following arterial injury.** (**A**–**C**) SM α-actin immunostaining (brown) of 2-week-injured femoral arteries. (**D**–**F**) Magnified area from boxed area of (**A**–**C**), respectively. Dashed lines demarcate media area. Arrows in (**E**) indicate areas lacking brown staining. med, media; neo, neointima. (**G**) Primary VSMCs were treated with IL-1β and 5-MTP and then Western performed to detect SM α-actin and CRP2. Quantitation of SM α-actin (**H**) and CRP2 (**I**) protein levels (n=3 each; *p<0.004 and ^#^*p*<0.002). (**J**) VSMCs were treated with TNF-α and 5-MTP and then Western performed. Quantitation of SM α-actin (**K**) and CRP2 (**L**) protein levels (n=3 each; *p<0.01 and ^#^*p*<0.01).

Phenotypic modulation of VSMCs regulates vascular remodeling. In response to injury, VSMCs change their phenotype from a differentiated, quiescent to a synthetic, proliferative, and migratory phenotype, and downregulate contractile markers such as smooth muscle (SM) α-actin and cysteine-rich protein 2 (CRP2) [28]. Given that *in vivo,* 5-MTP decreased injury-elicited VSMC proliferation and *in vitro*, 5-MTP reduced inflammatory cytokine-induced VSMC proliferation and migration, 5-MTP might maintain VSMCs in a differentiated phenotype after inflammatory cytokine stimulation. We thus examined the effects of 5-MTP on VSMC marker expressions by staining vessel sections with SM α-actin antibody. Compared with strong SM α-actin staining in the medial layer of uninjured arteries (Figure 7A and 7D), SM α-actin staining was notably decreased in the media and neointima 2 weeks after injury (Figure 7B and 7E). Intriguingly, 5-MTP group had smaller neointima and higher SM α-actin level (Figure 7C and 7F) than that from vehicle group, and comparable to that of uninjured. To investigate the underlying mechanisms, we treated primary VSMCs with inflammatory cytokines. IL-1β significantly reduced SM α-actin and CRP2 levels (Figure 7G–7I). Interestingly, 5-MTP rescued IL-1β-mediated downregulation of SM α-actin and CRP2 levels (Figure 7G-7I). TNF-α also had similar effects in reducing both SM α-actin and CRP2 levels (Figure 7J–7L) whereas 5-MTP rescued these VSMC markers (Figure 7J–7L). These data support that in response to inflammatory cytokine stimulation, 5-MTP mitigates VSMC marker gene downregulation and prevents VSMCs transitioning from a differentiated to a synthetic phenotype.

### 5-MTP rescues SRF and MRTF-A levels in VSMCs via suppressing NF-κB and p38 MAPK activation

**Figure 8:**
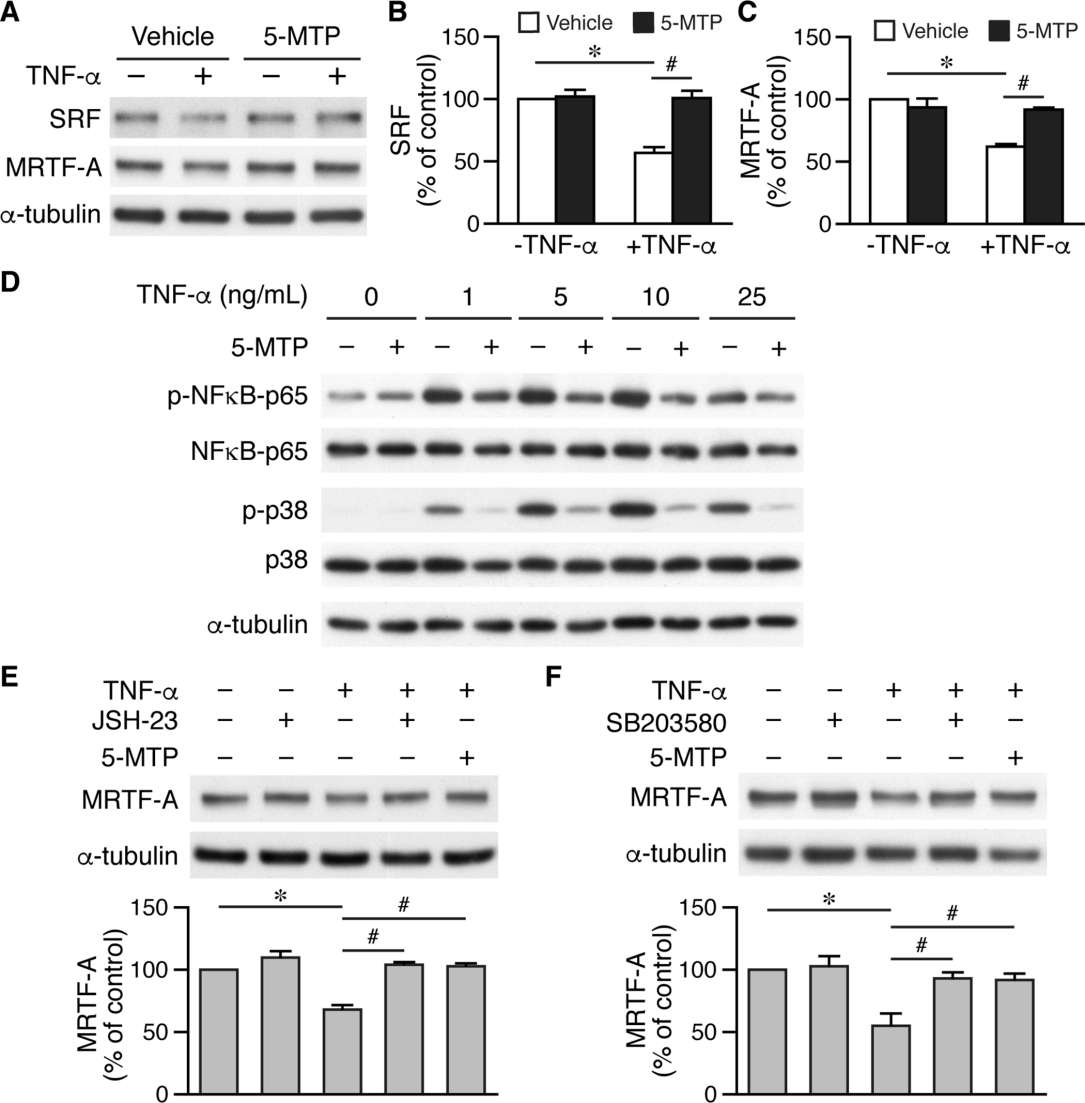
**5-MTP maintains critical transcription factor and cofactor levels in VSMCs via suppressing NF-κB and p38 activation.** (**A**) VSMCs were treated with TNF-α and 5-MTP for 24 h and Western performed to detect SRF and MRTF-A, and α-tubulin to verify loading. (**B**–**C**) Quantitation of SRF (**B**) and MRTF-A (**C**), n=3-4 each group, *p<0.0001 and ^#^*p*<0.002. (**D**) VSMCs were treated with TNF-α for 15 min and Western performed to detect phospho- and total NFκB-p65 and p38. A representative of 4 independent experiments is shown. (**E**) VSMCs were pretreated with NFκB inhibitor JSH-23 before TNF-α stimulation, followed by Western to detect MRTF-A (n=3 each, *p<0.002 and ^#^*p*<0.002). (**F**) VSMCs were pretreated with p38 MAPK inhibitor SB203580 before TNF-α stimulation, followed by Western to detect MRTF-A (n=3 each, *p<0.02 and ^#^*p*<0.04).

Smooth muscle markers are in general regulated by *cis*-element CArG box (CC(A/T)_6_GG) and cognate transcription factor serum response factor (SRF) and its cofactors, particularly myocardin and/or myocardin-related transcription factor-A (MRTF-A) [29]. TNF-α reduced SRF and MRTF-A levels to ~50% while 5-MTP rescued the downregulation of these two factors (Figure 8A–8C). TNF-α has been shown to activate NF-κB pathway and decrease myocardin level and VSMC differentiation markers [30]. Thus, we examined the role of 5-MTP in NF-κB activation. Interestingly, 5-MTP suppressed TNF-α-induced NF-κB activation (Figure 8D). To further confirm this finding, we pretreated cells with NFκB inhibitor JSH-23, followed by TNF-α stimulation. Western analysis revealed that JSH-23 blocked TNF-α-reduced MRTF-A expression and returned MRTF-A level while 5-MTP also abrogated TNF-α-reduced MRTF-A expression in VSMCs (Figure 8E). Because 5-MTP also inhibited TNF-α-induced p38 MAPK activation (Figure 8D), we tested whether p38 inhibitor SB203580 rescued TNF-α-reduced MRTF-A expression. Indeed, both SB203580 and 5-MTP ameliorated TNF-α-decreased MRTF-A expression (Figure 8F). These results suggest that 5-MTP might protect against injury-induced intimal hyperplasia by maintaining VSMCs in the differentiated phenotype through preserving critical VSMC transcription factors/cofactors via suppressing NF-κB and p38 activation in the injured arterial wall.

## DISCUSSION

In the present study, we demonstrated in an arterial denudation model that tryptophan metabolite 5-MTP protects against intimal hyperplasia via concerted but opposing actions on endothelial cells and VSMCs. A variety of metabolites derived from tryptophan metabolism play crucial physiological functions. Of the two major tryptophan metabolic pathways [13], the major kynurenine pathway (~95%) in mammals [31] is responsible for generating kynurenine and the related catabolites. Deregulated kynurenine pathway is linked to inflammation, oxidative stress, and immune activation in cardiovascular diseases [13, 31]. Increased plasma kynurenine/tryptophan ratio is positively correlated with CHD [32]. The second serotonin/melatonin pathway (~5%) of tryptophan metabolism generates critical metabolites such as neurotransmitter serotonin and circadian rhythm regulator melatonin [31]. Although serotonin is an important neurotransmitter, it is associated with CHD and cardiac events [33]. Interestingly, despite an unfavorable effect of serotonin in the cardiovascular system, cytoprotective 5-MTP is produced via a newly identified branch of serotonin/melatonin pathway, in which 5-hydroxytryptophan is converted to 5-MTP likely via hydroxyindole O-methyltransferase [13, 34]. It is conceivable that this new branch of pathway represents an endogenous defense mechanism and functions to divert tryptophan metabolism from production of serotonin to protective 5-MTP in the vasculature to counteract the damaging effects of serotonin.

Repairing endothelium is critical in inhibiting restenosis. Our results show that 5-MTP enhances reendothelialization after arterial denudation, likely due in part to increased endothelial cell proliferation and migration by 5-MTP that are mediated through VEGF/VEGFR2 activation. Although VEGF is also a vascular permeability factor, at the denuded arterial wall activation of VEGF/VEGFR2 signaling by 5-MTP may function mainly to increase endothelial cell proliferation and migration for restoring endothelial coverage rather than affecting permeability. A previous report shows that 5-MTP preserves HUVEC junctional protein VE-cadherin level and barrier function via p38 MAPK signaling despite challenges with inflammatory mediators [17]. Taken together, 5-MTP not only increases endothelial coverage on the denuded luminal surface but also functionally reduces vascular leakage.

Suppressing VSMC proliferation and migration after vessel injury is necessary in ameliorating vascular remodeling, which is in contrast to the favorable effects of endothelial proliferation and migration following injury. We found that in contrast to its effect on endothelial cells, 5-MTP significantly reduced injury/TNF-α-induced VSMC proliferation and migration. Given that proliferative and migratory properties are characteristics of the synthetic phenotype of VSMCs, 5-MTP might preserve differentiated phenotype of VSMCs. Indeed, 5-MTP maintained differentiated VSMC marker SM α-actin level in the injured arteries. Studies have shown that MRTF-A physically associate with SRF, facilitating the binding of SRF to CArG boxes on the SMC-restricted SM α-actin and SM22α promoters, leading to activation of gene transcriptions [35]. Further, we have shown that CArG boxes in the regulatory regions of the Csrp2 (gene name of the mouse CRP2) gene, together with SRF and MRTF-A regulate CRP2 transcription in VSMCs [36]. Previous findings and results from the present study support the idea that mechanistically, 5-MTP preserved critical transcription factors SRF and MRTF-A levels that are important in controlling VSMC marker gene expressions [29, 35]. Moreover, our data indicate that 5-MTP functions upstream of p38 MAPK and NFκB to block inflammatory cytokine-elicited signaling pathway activation, thereby maintains critical VSMC transcription factor/coactivator expressions, leading to preservation of VSMC marker levels and the differentiated phenotype. Interestingly, although 5-MTP appears to mediate its protective effect through p38 MAPK and NFκB signaling in VSMCs; in endothelial cells, 5-MTP exerts its protection primarily through p38 MAPK but not NFκB signaling.

**Figure 9:**
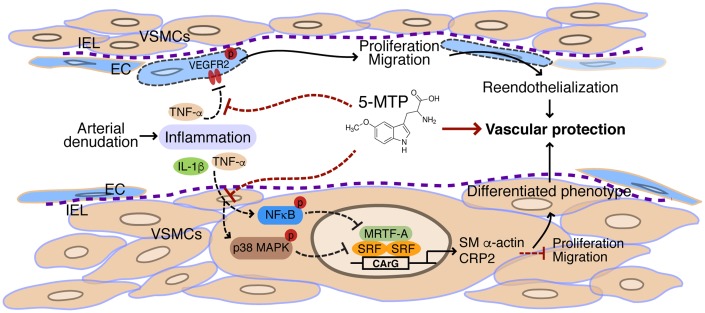
**Schematic illustration of vascular protective actions of 5-MTP.** In response to arterial denudation injury, inflammatory mediators IL-1β and TNF-α are released in the injured site. TNF-α inhibits VEGFR2 activation in endothelial cells (ECs). 5-MTP mitigates TNF-α-inhibited VEGFR2 phosphorylation, leading to EC proliferation and migration and consequent reendothelialization to protect blood vessels. In VSMCs, the inflammatory mediators activate NFκB and p38 MAPK pathways that lead to downregulation of transcription factor SRF and its cofactor MRTF-A, resulting in reduced expression of VSMC markers and converting to a synthetic phenotype of VSMCs. 5-MTP suppresses phosphorylation of NFκB and p38 MAPK, preventing downregulation of SRF and MRTF-A and sustaining expressions of VSMC markers. As such, 5-MTP inhibits VSMC proliferation and migration, and maintains differentiated phenotype of VSMCs. Ultimately, through combinatorial but opposing effects on ECs and VSMCs, 5-MTP protects against arterial injury-induced intimal hyperplasia.

In conclusion, we uncovered a novel protective mechanism of 5-MTP in restenosis. 5-MTP exerts its vascular protective effect following injury via concerted actions of enhancing reendothelialization and inhibiting migration and proliferation of VSMCs into intimal space (Figure 9). In response to arterial denudation injury, inflammatory mediators IL-1β and/or TNF-α released in the injured site inhibit VEGFR2 activation in endothelial cells and activate p38 MAPK and NFκB pathways in VSMCs. In endothelial cells, 5-MTP mitigates TNF-α-inhibited VEGFR2 phosphorylation, leading to endothelial cell proliferation and migration and consequent reendothelialization. In VSMCs, 5-MTP suppresses phosphorylation of p38 MAPK and NFκB, preventing downregulation of SRF and MRTF-A, which in turn sustains expressions of VSMC markers and maintains differentiated phenotype of VSMCs, resulting in attenuated VSMC proliferation and migration into intimal space. Ultimately, through combinatorial but opposing effects on endothelial cells and VSMCs, 5-MTP protects against arterial injury-induced intimal hyperplasia (Figure 9). Thus, 5-MTP may be a valuable lead compound to develop new drugs for preventing and treating stenosis/restenosis.

## MATERIALS AND METHODS

### Femoral artery denudation injury

The animals were housed at the National Health Research Institutes (Taiwan) animal facility. All experimental procedures were performed in accordance with NIH guidelines (Guide for the care and use of laboratory animals) and approved by the Institutional Animal Care and Use Committee of National Health Research Institutes, Taiwan (#NHRI-IACUC-105096-A). Approximately 12 weeks old male wild type C57BL/6JNarl mice (National Laboratory Animal Center, Taiwan) were subjected to a neointima formation model of femoral artery denudation injury as described [37]. Mice were treated with vehicle (PBS) or 5-MTP (25 mg/kg) by intraperitoneal injection the night before surgery. The next day, mice were anesthetized with isoflurane vapor by inhalation, 4-5% initially and 1-3% during the procedure, to achieve appropriate sedation. Endoluminal injury to the left common femoral artery was then performed to denude endothelium with 3-5 passages of a 0.014”-diameter guide wire (Hi-Torque Cross-it 100XT, with hydrocoat hydrophilic coating; Abbott, Abbott Park, Ill, USA). Following surgery, mice were treated with vehicle or 5-MTP 3 times a week until harvest [19].

### Histological analysis and immunohistochemistry

At indicated time points, mice were anesthetized with an overdose (500-750 mg/kg) tribromoethanol solution, perfused with PBS, followed by 10% neutral-buffered formalin (Sigma, St. Louis, MO, USA). The contralateral uninjured right common femoral artery (as control) and injured left common femoral artery were then carefully dissected, excised, and further fixed in 10% formalin at 4°C overnight before processing and embedding in paraffin. Serial 4-μm cross-sections or longitudinal sections of the femoral arteries were collected. Sections were stained with H&E or Verhoeff's stain (Sigma) for morphology and delineation of elastin layers, respectively. Three sets of sections at 150-μm (for cross sections) or 60-μm (for longitudinal sections) intervals were used for morphometry, the intimal and medial areas measured using NIH ImageJ software, and the intima-to-media ratio calculated essentially as described [19]. Vascular sections were immunostained with anti-CD31 antibody (ab28364; Abcam, Cambridge, MA, USA) to detect ECs. To assess endothelial coverage, CD31-positive luminal lining was divided by the total luminal lining and multiplied by 100. To determine cellular proliferation in the arterial wall, mice were injected with 50 mg/kg BrdU (Sigma, B9285) once at 16-18 h and the second time at 1-2 h before harvest. Vessel sections were stained with a BrdU antibody (M0744; Dako, Santa Clara, CA, USA) to detect proliferating cells and counterstained with hematoxylin. BrdU-positive cells were quantified and the number divided by total number of nuclei in the neointima or media, and expressed as percentage of total cells. To identify proliferating ECs, sections were double stained with CD31 (blue) and BrdU (brown) antibodies. To detect SM α-actin expression, sections were stained with anti-SM α-actin antibody (Sigma, A5528).

### Assessment of vascular leakage

To assess reendothelialization/barrier function of femoral arteries, 4 d and 1 week after injury mice were injected with 5% Evans blue (in PBS, Sigma) through tail vein 10 min before harvest, followed by 10% formalin perfusion via left ventricle [38]. The control and injured femoral arteries were then excised and opened longitudinally to expose the luminal surface. Blue area, indicative of vascular leakage, was quantified by NIH ImageJ software and vascular leakage determined by dividing blue area to total area.

### Endothelial cell culture, proliferation, and wound healing and transwell migration assays

HUVECs were purchased from Lonza (Tokyo, Japan) and cultured in endothelial basal media (Lonza), supplemented with endothelial growth media-2 (EGM-2) SingleQuot Kit (Lonza) containing defined supplements. Cells of passages 5-6 were used for experiments. Cells were grown in 24-well and 6-well plates for proliferation and wound healing assays, respectively. HUVECs were plated overnight and serum-starved in 0.5% FBS in endothelial basic medium (supplemented with only GA-1000, hydrocortisone, ascorbic acid, and heparin) in the presence of vehicle (DMSO) or 5-MTP (100 μmol/L) for 24 h. Cells were then incubated with different concentrations of human TNF-α (cyt-223; Prospec, East Brunswick NJ, USA) for 24 h. For proliferation assays, HUVECs were then stimulated with 50 ng/mL VEGF (Peproteh, Rocky Hill, NJ, USA) for 18 h and proliferation measured using Cell Counting Kit-8 (Dojindo Molecular Technologies, Rockville, MD, USA). For wound healing assays (to measure migration), the above serum-starved, treated cells in 6-well plates were wounded with a p200 tip and then incubated with or without 50 ng/mL VEGF for 18 h. Wound images were captured at time 0 and at 18 h. Wound closure was quantified by Image J software from 3 fields of each treatment. To rule out potential involvement of cellular proliferation in the wound closure, HUVECs were treated with mitomycin C (Sigma) to arrest growth, incubated with vehicle or 5-MTP for 24 h, placed in the upper chamber of 24-well transwell plate (8 μm pore size; Millipore, Taipei, Taiwan), and then stimulated with different doses TNF-α in the presence of vehicle or 5-MTP in triplicate (50,000 cells/well). EGM-2 medium containing 50 ng/mL VEGF was added into lower chambers as a chemoattractant. After 18 h, non-migrated cells were scraped from the upper side of membrane, membrane fixed, and stained with DAPI (Sigma). Cells that had migrated to the underside of the membrane were photographed by using a fluorescent microscope. Cell number was counted using x40 images and normalized to vehicle-treated control without TNF-α.

### Phosphorylation of vascular endothelial growth factor receptor 2 in HUVECs

To determine activation of VEGFR2, HUVECs were plated and treated as for the above proliferation/migration assays, and stimulated with VEGF (50 ng/mL) for 15 min. Total proteins were then isolated for Western blot analysis. To examine phosphorylation level of VEGFR2, blots were probed with phospho-VEGFR2 (Tyr1175) antibody (D5B11, #3770; Cell signaling, Danvers, MA, USA). To verify loading, blots were then probed with a pan-actin antibody (Millipore, MAB1501). The protein bands were quantified by Image J and normalized to vehicle control without TNF-α.

### VSMC culture, proliferation, migration, and viability assays

Primary VSMCs were isolated from mouse aortas and cultured in DMEM containing 10% FBS and passage 5-8 cells were used for experiments. To determine the effect of TNF-α on cell proliferation, VSMCs were plated in 24-well plates, serum starved (0.2% FBS) for 24 h and then treated with different concentrations of TNF-α along with BrdU for 24 h. Proliferation was then assessed according to manufacturer’s instructions (Millipore). To evaluate the effect of 5-MTP on TNF-α- or angiotensin II-induced VSMC proliferation, cells were plated, serum starved in the presence of vehicle DMSO or 5-MTP (100 μmol/L) for 24 h before treatment with different concentrations of TNF-α or angiotensin II for 24 h. Proliferation was then measured with BrdU incorporation assays. To assess migration, VSMCs were serum-starved in the presence of vehicle or 5-MTP (100 μmol/L) for 24 h, and treated with TNF-α (10 ng/mL) for 24 h. Cells were then placed in triplicate in the upper chamber of 24-well transwell plates (Millipore, 8-μm pore size; 10,000 cells/well). The bottom chambers were filled with starvation medium with or without 10 ng/mL PDGF-BB (Peprotech) as a chemoattractant. After 4 h, upper layer was scraped free of cells, membrane fixed, and stained with DAPI (Sigma). Cells that migrated to underside of the membrane were counted using x40 images and normalized to vehicle control without TNF-α treatment, and without PDGF-BB as a chemoattractant. For viability assays, VSMCs were plated and serum-starved in the presence of vehicle or 5-MTP as above and then treated with different doses of TNF-α for 24 h. Cell viability was determined by MTT assays (Sigma Aldrich, M2128). Cell viability was normalized to control without 5-MTP and TNF-α.

### Smooth muscle marker and transcription factor expressions in VSMCs

To evaluate expressions of VSMC markers and transcriptional regulators, VSMCs were plated and serum starved for 36 h in the presence of vehicle (DMSO) or 5-MTP (100 μmol/L), and then stimulated with or without mouse IL-1β (cyt-273, 10 ng/mL; Prospec) or mouse TNF-α (cyt-252, 10 ng/mL; Prospec) for 24 h. Total proteins were prepared using extraction buffer containing protease inhibitor Complete (Roche, Basel, Switzerland) for Western blot analysis. To detect SMC markers, membranes were probed with SM α-actin antibody (Sigma, A5528) and CRP2-(81-98) antibody (custom-made by GenScript, Piscataway, NJ, USA). Expressions of transcription factor SRF and its coactivator MRTF-A were detected by probing membranes with anti-SRF (Cell Signaling, #5147) and anti-MRTF-A (Cell Signaling, #14760) antibodies, respectively. The blots were subsequently probed with α-tubulin antibody (Cell Signaling, #3873) to verify equivalent loading. To examine signaling pathways, serum-starved VSMCs that had been treated with vehicle or 5-MTP for 36 h were stimulated with different concentrations of TNF-α for 15 min and total proteins prepared. Western blot analysis was then performed to detect p38MAPK and NFκB activations. Anti-p38 MAPK (Cell Signaling, #9212) and anti-phospho-p38 (Thr180/Tyr182; Cell Signaling, #9211) antibodies were used to detect total and phosphorylated p38 MAPK, respectively. Anti-NFκB p65 (Abcam, ab32536) and anti-phospho-NFκB p65 (Ser536; Cell Signaling, #3033) antibodies were used to detect total and phosphorylated NFκB p65, respectively. For loading control, blots were subsequently probed with α-tubulin antibody. To inhibit p38 MAPK or NFκB activities, VSMCs were pretreated with 30 μmol/L SB203580 (tlrl-sb20; InvivoGen, San Diego, CA, USA) or 20 μmol/L JSH-23 (Sigma, J4455) 30 min prior to stimulation with TNF-α, respectively.

### Statistics

Data are presented as mean ± S.E. of at least 3 independent experiments for *in vitro* studies. For *in vivo* studies, 4-13 mice per group were used for analysis. Data are analyzed by Student’s *t*-test. Statistical significance is considered at *p* value <0.05.

## Supplementary Material

Supplementary Figures
