# Supplementary material for: Tryptophan metabolite 5-methoxytryptophan ameliorates arterial denudation-induced intimal hyperplasia via opposing effects on vascular endothelial and smooth muscle cells
Source: Aging (Albany NY). 2019 Oct 9;11(19):8604–22. doi: 10.18632/aging.102350 (PMC6814618; doi:10.18632/aging.102350)
Supplement: Supplementary Figures [file aging-11-102350-s001.pdf]

SUPPLEMENTARY FIGURES

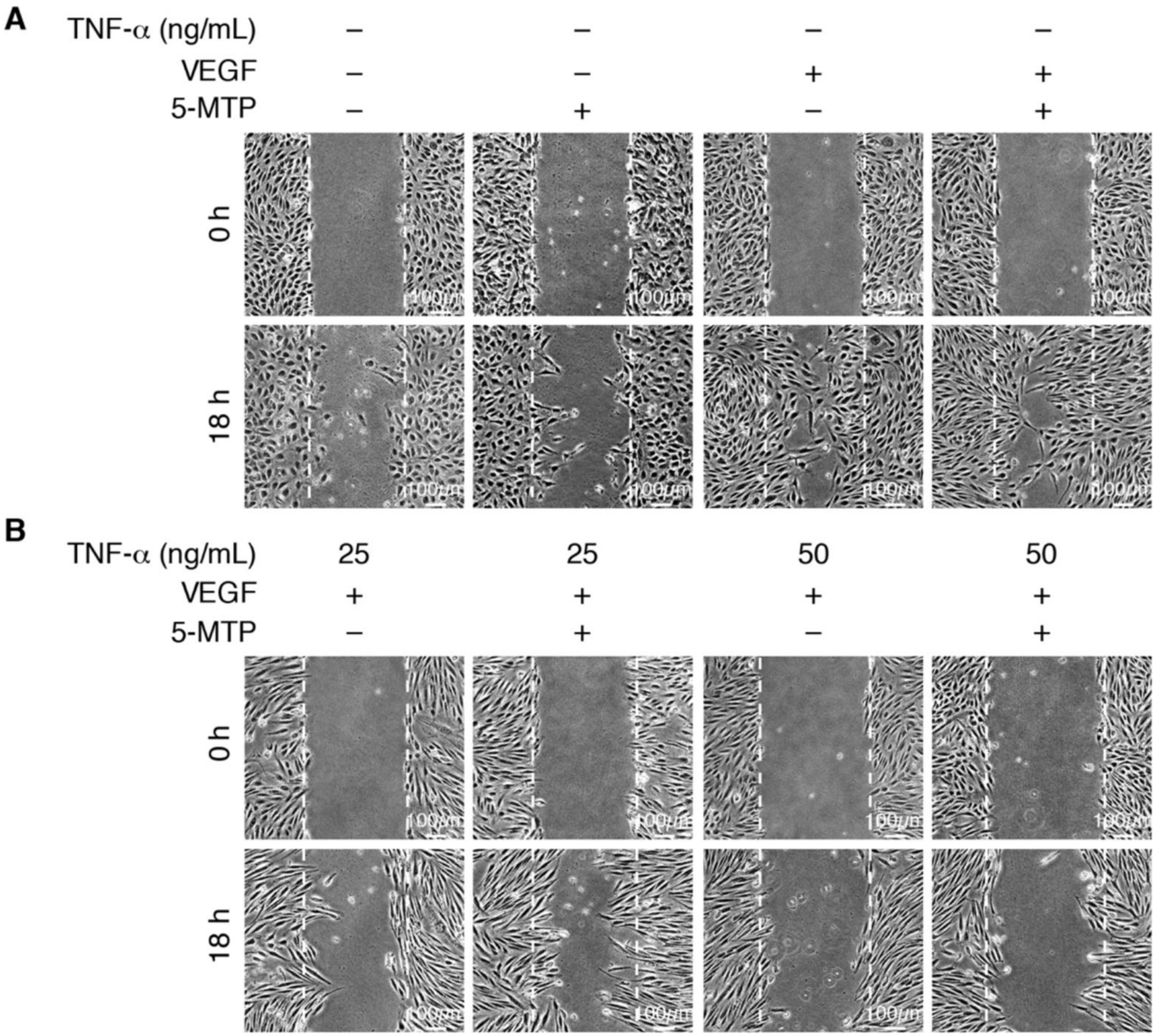

Supplementary Figure 1. 5-MTP rescues TNF-α-decreased endothelial cell migration. Serum-starved HUVECs were incubated with vehicle DMSO or 5-MTP before treatment with different concentrations of TNF-α (0, 25, 50 ng/mL) for 24 h. Cells were then wounded with a p200 tip and then stimulated with 50 ng/mL VEGF for cellular migration and wound closure. Photographs were taken at time 0 and 18 h after wounding. (**A**) Wound closure at 0 h and 18 h after wounding of cells without any treatment, 5-MTP only, VEGF only, or treated with VEGF and 5-MTP. Dashed lines demarcate wound margins. Representative images are shown. (**B**) Wound closure at 0 h and 18 h after wounding of cells treated with VEGF and TNF-α (25 or 50 ng/mL), in the presence or absence of 5-MTP. Dashed lines demarcate wound margins. Representative images are shown.

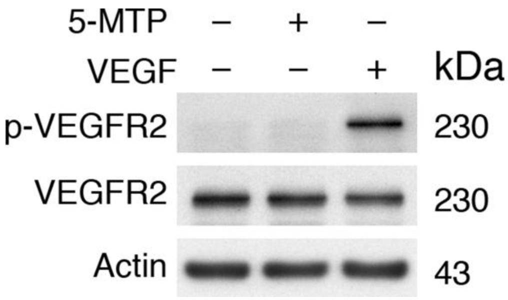

Supplementary Figure 2. 5-MTP does not affect VEGFR2 phosphorylation in endothelial cells without stimulation. Serum-starved FIUVECs were incubated with vehicle or 5-MTP for 24 h before stimulatation with or without 50 ng/mL VEGF for 15 min. Total proteins were then prepared for Western blotting to detect phosphorylated and total VEGFR2, and pan-actin for loading control.

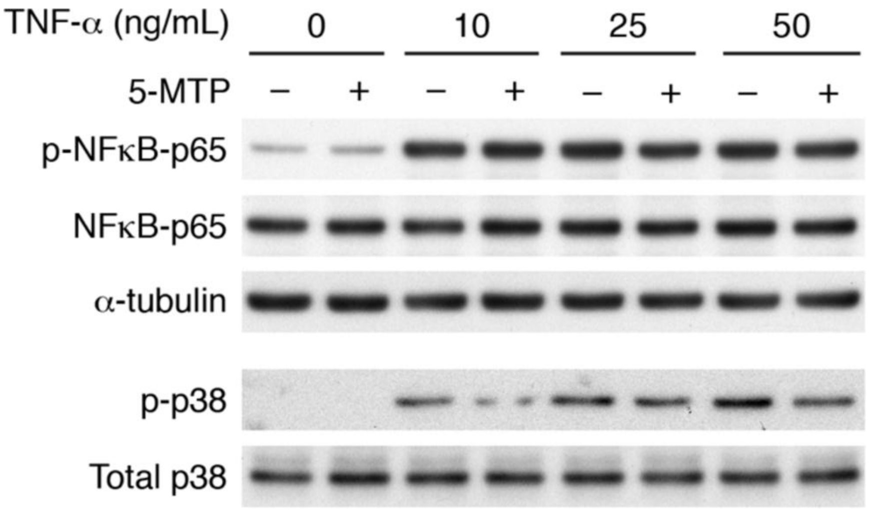

Supplementary Figure 3. 5-MTP suppresses TNF-α-inducted p38 activation but does not affect NFκB activation in endothelial cells. HUVECs were pretreated with vehicle or 5-MTP and then stimulated with different concentrations of TNF-α for 15 min. Western blotting was performed to detect phosphorylated and total p38 and NFκB-p65. To further verify equal loading, the blots were probed with a α-tubulin antibody. A representative of 3 independent experiments is shown.

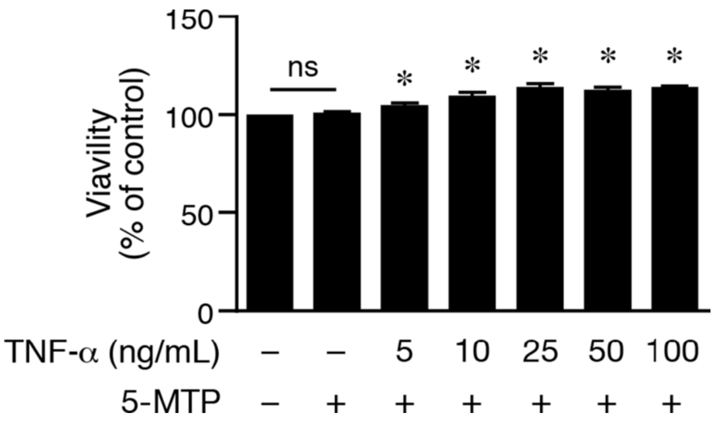

Supplementary Figure 4. 5-MTP does not affect VSMC viability in the absence or presence of TNF-α. Serum-starved VSMCs were incubated with vehicle or 5-MTP before stimulatation with or without TNF-α for 24 h. Cell viability was then determined by MTT assays. Cell viability was normalized to control without 5-MTP and TNF-α (set as 100%). n=3. ns, no significance; *p<0.02 vs. control.
